# The Role of Transthoracic Ultrasound in the novel Coronavirus Disease (COVID-19): A Reappraisal. Information and Disinformation: Is There Still Place for a Scientific Debate?

**DOI:** 10.3389/fmed.2020.00271

**Published:** 2020-05-27

**Authors:** Carla Maria Irene Quarato, Mariapia Venuti, Donato Lacedonia, Anna Simeone, Lucia Maria Cecilia Dimitri, Gaetano Rea, Beatrice Ferragalli, Marco Sperandeo

**Affiliations:** ^1^COVID-19 Center, Institute of Respiratory Diseases, Azienda Ospedaliero Universitaria Ospedali Riuniti di Foggia, Foggia, Italy; ^2^Department of Medical and Surgical Sciences, Institute of Respiratory Diseases, University of Foggia, Foggia, Italy; ^3^Unit of Radiology, COVID-19 Center, IRCCS Casa Sollievo della Sofferenza, San Giovanni Rotondo, Italy; ^4^Unit of Pathology, COVID-19 Center, IRCCS Fondazione Casa Sollievo della Sofferenza, San Giovanni Rotondo, Italy; ^5^Radiology Section, Department of Imaging, Monaldi Hospital, Naples, Italy; ^6^Unit of Radiology, Department of Medical, Oral and Biotechnological Sciences, Adriatic University “G d'Annunzio”, Chieti, Italy; ^7^Unit of Interventional and Diagnostic Ultrasound of Internal Medicine, COVID-19 Center, IRCCS Fondazione Casa Sollievo della Sofferenza, San Giovanni Rotondo, Italy

**Keywords:** transthoracic ultrasound, ultrasound artifacts, COVID-19, diagnostic accuracy, lung imaging

An human SARS-like coronavirus (SARS-CoV-2) has spread globally, resulting in the novel Coronavirus disease 2019 (COVID-19) pandemic. Given the high contagiousness of SARS-CoV-2 and the seriousness of COVID-19, there is currently great pressure on the scientific community to provide answers for the diagnosis and treatment of such a disease. However, the diagnostic accuracy of imaging methods and the effectiveness of treatments take time to prove and the dissemination of premature conclusions may result in a misdiagnosis and malpractice.

In particular, recent works in the literature have highlighted the possibility that a transthoracic ultrasound examination of the lung, not only allows one to make a diagnosis of SARS-CoV-2 pneumonia, but also provides the possibility of following up on the patient during therapy, with a very high diagnostic accuracy, comparable to that of a chest CT. Any scientific journal has the possibility, if not the duty, to allow a scientific comparison of what has previously been published, where doubts and/or strong perplexities are detected. Despite this, we have previously tried to raise our doubts about the recent widespread and improper use of thoracic ultrasound for the diagnosis of COVID-19 and this opportunity has been repeatedly denied to us. For this reason, we are pleased to provide an overview of our perplexities in this journal.

## Can a Transthoracic Lung Ultrasound Pattern be Considered Specific for COVID-19?

Recently Peng et al. (1) claimed that a lung ultrasound is useful for a rapid assessment of the severity of SARS-CoV-2 pneumonia/ARDS at presentation and during follow-up; chest CT may be reserved for cases where this imaging method is not sufficient to answer clinical questions. The most important ultrasound sign in the early stage and in a mild infection would be focal B-lines, while an “alveolar interstitial syndrome” (i.e., a pattern of multifocal and confluent B-lines) is considered to be the main feature in the progressive stage and in critically ill patients. This appears to suggest that B-lines have gained widespread scientific acceptance as a marker of “interstitial edema”, but, to our knowledge, no approved international recommendation/guideline reports this indication.

“B-lines,” are only artifacts generated by the physical interaction between the ultrasound beam and the different structures crossed by it. Indeed, we cannot find them during an intraoperatory pathological lung ultrasound (ILU) examination, a technique in which the ultrasound probe is directly placed on the lung (2). In particular, B-lines artifacts originate from microbubbles of air/gas, mixed with liquid film/edema and/or fibrosis, which resonate with the ultrasound beam (3).

Several problems affect the reliability of the diagnostic use of B-lines.

First, their specificity is suboptimal: in addition to pulmonary congestion/ARDS, B-lines are visible in: heart failure, nephrotic syndrome, pneumonia, minimal pleural effusion, hydropneumothorax, fibrosis, emphysema, exacerbations of chronic obstructive pulmonary diseases, and lymphangitis (4–6). Few B-lines are observable even in a healthy lung, typically in the dependent regions, and in the post-pneumonectomy space (7).

Second, the evaluation process of B-lines is perceptive semi-quantitative, because the method is more of a subjective overview than an actual “measurement.” The simple change of positioning of the probe, with respect to the curvature of the patient's chest, can modify the perception of B-lines. To obtain a “valid” estimate of B-lines, the physician has to “freeze” the LUS image, count the lines, and repeat it every time the probe position is changed (8). It is very difficult to think that it would be possible to apply this technique for the assessment of suspected COVID-19 patients, a condition in which it is better not to prolong the examination in order to reduce the risk of infection. The increase in the pleural line movement rate in dyspneic patients can also modify the perception of B-lines (8). Nevertheless, there is still not a standardized consensus on the ultrasound scan machine setting, as well as the type and frequency of the probe, which have to be used to perform a transthoracic ultrasound examination of the lung. The use of a medium-to-low frequency or excessive total gain (>50%) and the lack of tissue harmonic imaging can generate a larger number of ultrasound artifacts and this may result in another source of bias (9). Despite this, none of these recent articles, exalting the role of a lung ultrasound in the diagnosis of COVID-19, specify the setting of the ultrasound equipment employed.

Third, the perceptive semi-quantification of B-lines alone does not make any significant contribution to the differential diagnosis or prognostic assessment of a specific disease (10). Moreover, the same comorbidity conditions eventually present in subjects affected by SARS-CoV-2 pneumonia (mainly elderly patients) can give rise to confusion. In this initial period of virus spread, an ultrasound diagnosis based on these signs is more likely to be “statistically” correct, but when the incidence of this pneumonia becomes stable in the population, the risk of false positives and consequent misdiagnoses will increase.

## Ultrasound vs. Other Imaging Methods in the Diagnosis of COVID-19

**Figure 1:**
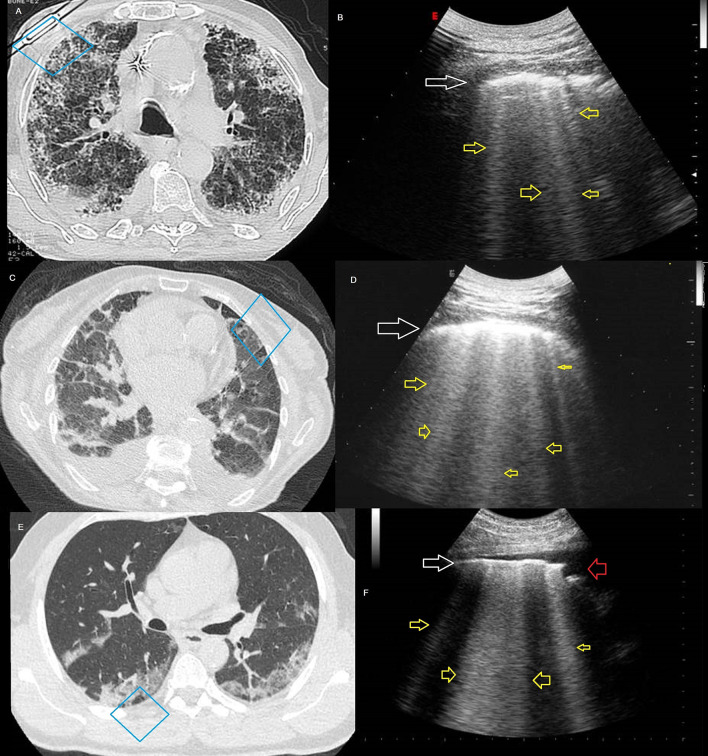
**(A)** Axial high resolution (HR) CT imaging showing a pattern of non-specific interstitial pneumonia (NSIP). **(B)** Ultrasound scan (corresponding to the blue box in the A CT scan), with convex probe (6 MHz) and thoracic setting showing irregular thickening of hyperechoic pleural line (white arrow) and B-lines below (yellow arrows). **(C)** Axial CT imaging showing signs of interlobular interstitial pulmonary edema. **(D)** Ultrasound scan (corresponding to the blue box in the C CT scan), with convex probe (6 MHz) and thoracic setting showing irregular thickening of hyperechoic pleural line (white arrow) and B-lines below (yellow arrows). **(E)** Axial CT imaging, in a patient with a fever for 1 week and positive results of RT-PCR assay for the SARS-CoV-2, showing bilateral peripheral ground-glass opacity associated with smooth interlobular and intralobular septal thickening. **(F)** Ultrasound scan (corresponding to the blue box in the E CT scan), with convex probe (6 MHz) and thoracic setting showing irregular hyperechoic pleural line (white arrow), striated subpleural hypoechogenicity (red arrow), and B-lines below (yellow arrows).

Similarly to Peng et al. (1), Poggiali et al. (11) suggested the use of “a diffuse B-pattern with spared areas” (i.e., presence of numerous B-lines with spared areas) on an ultrasound, for the early diagnosis of COVID-19 in emergency departments, affirming that ultrasound is “a highly sensitive and specific technique considered as an alternative to chest radiography or CT scanning” (11). Due to the hindrance of the thoracic cage and the lung air content however, only 70% of the pleural surface can be explored by ultrasound (12) and only the peripheral adherent to the pleura processes can be assessed, too small a part of the total lung parenchyma to study a widespread disease such as COVID-19 and/or ground glass or consolidation areas not adherent to pleural surface (13–15). The CT features of early-stage COVID-19 include ground glass opacities (GGOs)-based lesions with rare small size consolidation mainly distributed in the peripheral and posterior part of the lung. Some patients' pulmonary lesions are small and focal (16). However, not all the CT consolidation areas of pneumonitis are always adherent to the 70% of the superficial pleura or, is even accessible to the ultrasound beam. Likewise, the deeper CT GGOs-based lesions cannot be sonographically diagnosed on the basis of an ultrasound pattern of artifacts, such as the presence of a thickened hyperechoic pleural line with B-lines below, which is also common in many other lung diseases (i.e., pulmonary fibrosis or acute pulmonary edema) (17). (Figure 1) As a result, there is a risk of missing the detection of some lesions and/or to underestimate the actual disease's extent. That said, why not also perform at least a portable Chest X-ray, better if exclusively for COVID-19 patients, in the antero-posterior projection only (accessible in any emergency department)? This would allow us to assess the global involvement of the lung fields and the presence of mediastinal and cardiovascular comorbidities, if any.

To these considerations we have to add that a bedside ultrasound is the imaging investigation that involves the most interaction between a doctor and patient, therefore, in the case of indispensable and indifferent tests, to be performed in patients with COVID19, the SIUMB, SIRM, and FISM guidelines recommend, for general ultrasound, to practice the appropriate and complete dressing, using all personal protective equipment (PPE) necessary for this type of contact. The ultrasound probe must be cleaned with the appropriate sprays or disinfectants before and after use, and when possible or necessary, be covered with disposable plastic film^1^. In this context, performing a Chest X-ray at the patient's bed would seem to be the most practical choice. Other recommendations (ACR, BTS, ERS) do not suggest ultrasound examination on COVID-19 patients at all.

In another article, Buonsenso et al. (18) even suggested the use of an ultrasound pocket device consisting of a probe and a tablet protected by disposable removable covers for the execution of lung examination at the COVID-19 patient's bedside, reducing health-care workers' risk of exposure. However, the use of US is, by nature, imprecise as it depends on both the resolution of the image and on the operator. No international consensus has been reached on the empirical use of ultrasound in the management of COVID-19 in the multivariable context of respiratory disease's severity, pre-test probability, risk factors for disease progression, and critical resource constraints. In addition pocket-size imaging devices, currently used especially in echocardiography, are only screening tools and shouldn't be used for a complete echographic examination (19). Moreover, the same Chest CT, which tries to volumetrically quantify the lung parenchymal involvement in pneumonia, is not able to define the etiology of the disease with certainty. Indeed the findings on chest imaging in COVID-19 overlap with other viral infections, including influenza, metapneumovirus, and adenovirus (14, 20, 21). Therefore, the Fleischner Society and the Centers for Disease Control (CDC) recommend confirmation with the viral test, even if radiologic findings are suggestive of COVID-19 on CXR or CT (21, 22). How could we think of diagnosing such disease using only a transthoracic lung ultrasound examination?

## Discussion and Conclusion

With these concerns in mind, we believe that transthoracic ultrasound lung examination should not be considered a substitute examination in SARS-COV-2 pneumonia and it is not the time for the use of an ultrasound “diagnostic” pattern based on unspecific artifacts. Indeed, the reported signs are common to many pathologies and the frequent comorbidities of COVID-19 patients does not allow transthoracic lung ultrasound to be a decisive investigation (Table 1). Transthoracic lung ultrasound is only able to assess if the pleura and lung are abnormal in 70% of the observable surface, but it never defines the disease's etiology. This imaging tool may be considered an accurate examination only in pleural effusion, a rare finding in SARS-COV-2 pneumonia. The spreading of the idea that ultrasound is the most economical and autonomous solution to discriminate patients with lung involvement from COVID-19 is misleading and potentially dangerous. In this time of pandemic, we need a scientifically shared diagnosis. After a positive result from viral tests, to perform at least a chest X-ray represents a better choice in the initial definition of COVID-19, leaving the chest CT—despite perhaps being less practical to execute—the gold standard in the assessment of its extent. For the definition of the disease's gravity and its follow-up, we believe that the measurement of a quantitative variable, such as saturation and/or PCR value (21), is more reliable than a scarcely reproducible perceptive measurement technique, such as ultrasound counting of B-lines, also when considering the higher risk of contamination linked with the ultrasound examination. The scientific community has the duty to avoid the dissemination of erroneous information.

**Table 1:** Limits and risks of ultrasound use in COVID-19.

**Ultrasound findings in COVID-19**	**Limits and risks**
Increased number of focal B-lines coalescent or not (early stage and mild infection)	• **Perceptive semi-quantitative evaluation process:** the change of positioning of the probe with respect to the curvature of the patient's chest and the pleural line movement rate in dyspneic patients can modify the perceptive number of B-lines in real time ultrasonography
	• **Ultrasound scan machine setting:** the use of a medium-to-low frequency or excessive total gain (>50%) and the lack of tissue harmonic imaging can generate a larger number of ultrasound artifacts
	• **False positive conditions:** ARDS, heart failure and pulmonary edema, nephrotic syndrome and severe chronic renal failure, pneumonia, as well as minimal pleural effusion, hydropneumothorax, fibrosis, emphysema, exacerbations of chronic obstructive pulmonary diseases, pulmonary contusion, and lymphangitis
	• **Risk:** misdiagnoses due to comorbidity conditions eventually present in subjects affected by SARS-CoV-2 pneumonia (mainly elderly patients)
Thickened hyperechoic pleural line with B-lines below (intermediate-progressive stage)	• **False positive conditions:** pulmonary fibrosis, bronchiectasis, exacerbations of chronic obstructive pulmonary diseases, acute pulmonary edema, subpleural panlobular emphysema, subpleural pulmonary bullae, blebs, and cystic air spaces.
	• **Risk:** comorbidity conditions eventually present in subjects affected by SARS-CoV-2 pneumonia can give rise to confusion
Consolidation mainly distributed in the peripheral and posterior part of the lung	• **Limited assessment:** only 70% of the pleural surface and exclusively the lesions adherent to pleural surface can be explored by ultrasound
(progressive and late stage)	• **Risks:** (1) to miss the detection of deeper lesions and/or underestimate the actual disease's extent; (2) misdiagnoses due to comorbidity conditions eventually present in SARS-CoV-2 patients: lung cancer, non-viral pneumonia, other viral pneumonia, atelectasis and other consolidations

## Author Contributions

All authors have contributed to the conception and the writing of the paper, approving its final version.

## Conflict of Interest

The authors declare that the research was conducted in the absence of any commercial or financial relationships that could be construed as a potential conflict of interest.

## Footnotes

^1^COVID-19 SIUMB INFORMA | SIUMB. Available online at: http://www.siumb.it/?q=node/646.
